# Guillain-Barré syndrome and SARS-CoV-2

**DOI:** 10.1186/s42466-020-00066-0

**Published:** 2020-07-08

**Authors:** Anne Lampe, Alexander Winschel, Cornelie Lang, Thorsten Steiner

**Affiliations:** 1 Department of Neurology, Klinikum Frankfurt Höchst, Frankfurt am Main, Germany; 2 Department of Neurology, University Hospital Heidelberg, Heidelberg, Germany

**Keywords:** Guillain-Barré syndrome, COVID-19, SARS-CoV-2

## Abstract

Since January 2020, after Chinese health authorities identified a new type of coronavirus (SARS-CoV-2), the virus has spread throughout China and consecutively throughout the whole world. The most common symptoms include fever and respiratory tract symptoms. Nevertheless, some patients show less common symptoms such as gastrointestinal or neurological manifestations. This article presents the case of a 65-years old man who was presumptively infected with SARS-CoV-2 during his ski vacation in Austria in March 2020 and acutely presented with typical symptoms of Guillain-Barré syndrome.

## Introduction

In December 2019 multiple cases of atypical pneumonia were reported in Wuhan, China. A few weeks later the unkown pathogen was identified as a new type of coronavirus (SARS-CoV-2) by Chinese health authorities. The disease caused by SARS-CoV-2 was named coronavirus disease 2019 (COVID-19), which has spread rapidly from Wuhan throughout the whole world.

SARS-CoV-2 is a single-stranded RNA virus and belongs to the family of beta coronaviruses [1]. Coronaviruses are capable of causing illnesses in humans ranging from mild respiratory infections to more severe diseases such as severe acute respiratory syndrome (SARS) and Middle East respiratory syndrome (MERS) [2, 3].

The most common symptoms of COVID-19 are fever, dry cough and abnormal fatigue [4]. Beyond that, SARS-CoV-2 is not limited to the respiratory tract causing a wide range of clinical manifestations. A growing body of evidence shows that SARS-CoV-2 may also invade the central nervous system, thus inducing neurological diseases [5]. For example, many patients report headache, anosmia and ageusia [6]. There is an ongoing debate whether respiratory distress is not exclusively the result of a pulmonary inflammatory process, or whether a possible neuroinvasion by SARS-CoV-2 into the brainstem may play a role [7]. Moreover, neurological complications caused by coronaviruses were already reported during the outbreaks of SARS-CoV in 2002 and MERS-CoV in 2012 [8, 9].

The range and pathogenesis of neurological manifestations of SARS-CoV-2 are still largely unknown. We herein report about a COVID-19 patient who was admitted to the emergency department of our hospital with typical symptoms of Guillain-Barré syndrome (GBS).

## Case presentation

On March 10th 2020, a 65-years old man presented with a 2-day history of acute weakness of his right arm and lower limbs, which caused recurrent falls. He had no past medical history and was a nonsmoker. In mid-February he had already suffered from mild respiratory symptoms, which successfully resolved after an oral antibiotic treatment. After returning from his ski vacation in Austria he developed fever (38.0 °C) and a dry cough on March 7th 2020.

The neurological examination revealed a distally accentuated paresis of the right arm and a slight paraparesis of the lower limbs which was more pronounced on the right side. He had no sensory deficits. Deep tendon reflexes were reduced generally. The patient was afebrile (37.4 °C), had no dyspnea and the oxygen saturation was 98%.

Cerebrospinal fluid (CSF) analysis showed a slight increase in protein level (56 mg/dl) with a normal cell count (2 cells/μl). The laboratory tests also showed a slightly increased CRP of 1.92 mg/dl. In addition, ganglioside antibodies (GM1, GM2, GM3, GD1a, GD1b, GT1b, GQ1b) were tested and found to be negative. Summarizing all symptoms, we diagnosed GBS and initiated an intravenous immunoglobulin (IVIG) treatment (0.4 g/kg bodyweight per day for 5 days).

On the following day a progression of the right arm paresis and areflexia were recognized. In addition, the blood tests showed a slight leukopenia (3.0 /nl). Electrophysiologic tests revealed prolonged distal motor latencies of the right median and tibial nerves as well as increased F-wave latencies of the median and tibial nerves on both sides, consistent with a demyelinating polyradiculoneuropathy.

Because of persistent cough and fever, a chest x-ray was performed, which showed no conspicuous findings. Influenza and respiratory syncytial virus infection were excluded by polymerase chain reaction (PCR). Due to an increasing number of SARS-CoV-2 infections in ski resorts in Austria were reported and Tyrol was officially declared a high-risk area on March 14th 2020, we performed a throat swab test and subsequent PCR for SARS-CoV-2 which was positive. In consequence, a pathogen-suitable isolation was initiated.

Treatment with IVIG for a total of 5 days in combination with physiotherapy quickly led to a significant improvement in GBS symptoms. Hence, the patient could be discharged from the hospital 12 days after admission. At the time of discharge, there was no residual paresis while the deep tendon reflexes were still absent except for the left biceps reflex. The patient was also free of fever and the cough had significantly decreased. Fortunately, no infections occurred in the medical staff as a result of strict isolation measures in accordance with the most recent recommendations of the Robert Koch Institute.

## Discussion

The symptoms of COVID-19 range from mild unspecific symptoms to severe lower respiratory tract symptoms with pneumonia, acute respiratory distress syndrome (ARDS) and multiple organ failure [10]. There are numerous reports about the neuroinvasive potential of coronaviruses and an association between GBS and coronavirus infections [9, 11, 12].

GBS is an acute immune-mediated, demyelinating polyradiculoneuropathy characterized by ascending flaccid paralysis and reduced or absent muscle reflexes. GBS usually occurs 2 to 4 weeks after a respiratory or digestive tract infection. Typical pathogens include Campylobacter jejuni, Mycoplasma pneumoniae, Epstein-Barr-Virus, Cytomegalovirus and Zika virus [13]. The interval between the onset of symptoms of SARS-CoV-2 infection and first symptoms of GBS was only 1 day in the case presented here. Due to the chronological sequence, a causal relationship can be assumed. Nevertheless, it cannot be ruled out that GBS may have been caused by the respiratory infection 4 weeks earlier, which had been successfully treated with antibiotics**.** A case study of 5 patients with SARS-CoV-2 infection and GBS was published a few weeks ago [14]. In this series the first neurological symptoms also appeared already 5 to 10 days after the onset of the virus infection. Another case report indicates a simultaneous occurrence of COVID-19 and GBS. Based on this observation, the authors discussed a coincidence as well as a parainfectious pattern as already observed in GBS associated with Zika virus [15, 16]. This is in contrast with the typical postinfectious pathogenesis of GBS. Possible explanations are that direct viral neuropathogenic mechanisms may play a role or that an autoimmunological process is induced before clinical symptoms of COVID-19 occur. The latter is supported by a mean incubation period of 5 days up to a maximum of 14 days and the early onset of infectivity (2.5 days before onset of symptoms) [17, 18].

In our case, a decrease in leukocytes was noted as described for COVID-19 [4]. GBS is an immune-mediated disease in which cross-reactions with myelin and ganglioside antigens may play a central role in the context of molecular mimicry [13]. Ganglioside antibodies were not detectable in our patient. It is not yet known whether SARS-CoV-2 may induce the formation of specific antibodies against gangliosides.

Overall, both diseases, GBS and COVID-19, were mild in the patient presented here. There was no loss of walking ability, no impairment of the autonomic nervous system or a reduction of pulmonary vital capacity. Furthermore, rapid recovery was achieved under therapy with IVIG. At present, there is no vaccine or specific antiviral therapy for COVID-19. Therefore, this immunotherapy, which has been established in the treatment of autoimmune and inflammatory diseases for many years, is currently evaluated as an adjuvant treatment of COVID-19. First results suggest that IVIG and especially IVIG collected from recovered COVID-19 patients have the potential to neutralize SARS-CoV-2, thus posing a therapeutic option for critically ill patients [19, 20].

One important limitation of this case report is the delayed testing for SARS-CoV-2 on the 5th day after admission to hospital due to recommendations of the Robert Koch Institute at that time. Another limitation is the lack of PCR for SARS-CoV-2 in CSF.

There are still only a few reports of the neurological complications of SARS-CoV-2 since the global outbreak a few months ago. The present case underlines the importance to be aware of possible neurological manifestations of COVID-19. Moreover, it substantiates the evidence of a potential association between SARS-CoV-2 and GBS. It is also consistent with other publications which demonstrated similar chronological patterns of neurologic symptoms which differ from the classical postinfectious presentation of GBS. However, additional studies are necessary to further analyse and confirm these findings.

## Data Availability

The data that support the findings of this study are available from the corresponding author, upon reasonable request.

## Abbreviations

SARS: Severe acute respiratory syndrome
MERS: Middle East respiratory syndrome
GBS: Guillain-Barré syndrome
CSF: Cerebrospinal fluid
CRP: C-reaktive protein
IVIG: Intravenous immunoglobulin
PCR: Polymerase chain reaction
ARDS: Acute respiratory distress syndrome
